# A Pantropical Analysis of Fire Impacts and Post‐Fire Species Recovery of Plant Life Forms

**DOI:** 10.1002/ece3.71018

**Published:** 2025-02-17

**Authors:** Dharma P. Sapkota, David P. Edwards, Mike R. Massam, Karl L. Evans

**Affiliations:** ^1^ Ecology and Evolutionary Biology, School of Biosciences University of Sheffield Sheffield UK; ^2^ Department of Plant Sciences and Centre for Global Wood Security University of Cambridge Cambridge UK; ^3^ Conservation Research Institute, University of Cambridge Cambridge UK

**Keywords:** biodiversity, conservation, fire management, flora, species richness, species turnover

## Abstract

Fires are a key environmental driver that modify ecosystems and global biodiversity. Fires can negatively and positively impact biodiversity and ecosystem functioning, depending on how frequently fire occurs in the focal ecosystem, but factors influencing biodiversity responses to fire are inadequately understood. We conduct a pan‐tropical analysis of systematically collated data spanning 5257 observations of 1705 plant species (trees and shrubs, forbs, graminoids and climbers) in burnt and unburnt plots from 28 studies. We use model averaging of mixed effect models assessing how plant species richness and turnover (comparing burnt and unburnt communities) vary with time since fire, fire type, protected area status and biome type (fire sensitive or fire adaptive). Our analyses bring three key findings. First, prescribed and non‐prescribed burns have contrasting impacts on plant species richness (trees/shrubs and climbers); prescribed fire favours increased species richness compared to non‐prescribed burns. Second, the effect of time since fire on the recovery of species composition varies across all life form groups; forb's species composition recovered faster over all life forms. Third, protection status alters fire impacts on the species richness of trees/shrubs and climbers and species recovery of graminoids. Non‐protected areas exhibit higher species richness compared to protected areas in trees/shrubs, and climbers. Graminoid species composition recovered quicker in protected sites compared to unprotected ones. Since fire intervals are decreasing in fire‐sensitive biomes and increasing in fire‐adaptive biomes, plant communities across much of the tropics are likely to change in response to exposure to fire in the future.

## Graphical abstract

Fires are key environmental drivers that alter ecosystems and biodiversity. Depending on the type, intensity and frequency, they can impact biodiversity and ecosystem functioning positively or negatively, though the factors influencing these responses are poorly understood. Our three key findings reveal that: (1) Prescribed burns enhance species richness in trees/shrubs and climbers compared to non‐prescribed burns; (2) time since fire affects species composition recovery differently across life forms, with forbs recovering faster; (3) protection status modifies fire effects, showing higher species richness in non‐protected areas for trees/shrubs and climbers, while graminoids recover quicker in protected sites. As fire intervals decrease in fire‐sensitive biomes and increase in fire‐adaptive ones, plant communities in tropical regions are expected to change in response to fire exposure.

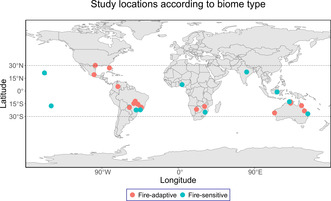

## 1 Introduction

Globally, the distribution, seasonality, frequency and intensity of fires have changed in recent decades due to anthropogenic global change drivers, including climate change, land‐use change (with fire often used to clear vegetation to facilitate land‐use change) and, in some cases, invasion by more flammable species (McLauchlan et al. 2020; Kelly et al. 2020). These changes are predicted to accelerate over the next few decades (Sheehan et al. 2019; Enright et al. 2015; Aragão et al. 2008). There is particular concern regarding the impacts on fire‐sensitive tropical ecosystems, many of which are being rapidly lost and degraded (Alroy 2017; Busch and Ferretti‐Gallon 2017), making the tropics the epicentre of current and future extinction risk (Edwards et al. 2019). Given these changing fire regimes, it is of utmost importance to understand how fire influences biodiversity and the recovery rate following fire events (Kelly et al. 2020). For example, this need is widely recognised by the Intergovernmental Science‐Policy Platform on Biodiversity and Ecosystem Services (IPBES 2019) and the UNFCC REDD+ program (UNFCC 2019).

Fire's impacts on biodiversity are complex and incompletely understood (Gill et al. 2013; McLauchlan et al. 2020; Tingley et al. 2016). Positive and negative impacts have been reported depending on the ecosystem, fire return interval, recovery time, and plant traits (Kelly et al. 2020; Giorgis et al. 2021; Driscoll et al. 2010). Moreover, fire, along with other disturbances such as herbivory by large vertebrates, serve a significant role in creating a dynamic ‘alternative stable state’ between forests and grasslands/savannas (Dantas et al. 2016; Pausas and Bond 2020). Despite sharing similar climate and soil conditions, two ecosystems, one open and the other closed, can develop in close proximity due to differing levels of exposure to fire (Dantas et al. 2016; Hoffmann et al. 2012; Charles‐Dominique et al. 2015). However, these ecosystems can shift in either direction depending on the level of exposure to fire and human disturbances (Pausas and Bond 2020; Williamson et al. 2024).

Fire‐adaptive ecosystems, such as tropical savannas and grasslands, are frequently exposed to fire, and several species characteristic of these biomes require fires to persist (Simon and Pennington 2012). In such biomes, fire positively influences the diversity of photophilic floras and faunas (Pausas and Keeley 2019), with a landscape mosaic of vegetation patches that vary in the time since they were burnt typically maximising biodiversity (Driscoll et al. 2010). Long‐term suppression of fire in these systems typically generates more homogenous vegetation patches that support fewer species (Giorgis et al. 2021; Abreu et al. 2017), promotes woody species and gradual shifts from grasslands to woody savannas, and then shrublands and forests (Probert et al. 2019).

In contrast, fire is historically extremely rare in fire‐sensitive biomes, such as tropical moist and other closed forests, and most plant species are highly sensitive to fire (Cochrane and Schulze 1999; Giorgis et al. 2021). Consequently, recent increases in the number of fires are a primary driver of tropical moist forest degradation and biodiversity loss (Barlow et al. 2019; Lewis et al. 2015; Cochrane and Schulze 1999). Increased exposure to fire can also eventually convert moist tropical forest ecosystems into open habitats and savannas (Flores and Holmgren 2021).

The influence of protected areas on biodiversity recovery following fire is also insufficiently understood (Pereira et al. 2012; Eklund et al. 2022; Kearney et al. 2020). Species recovery rates are likely to be faster within relatively intact ecosystems, that is effectively protected from anthropogenic stressors (Aide et al. 2012) in which a greater abundance of natural vegetation increases the availability of propagules that can recolonise burnt sites (Shepherd et al. 2021; Holl et al. 2000). However, the role of propagule dispersal from unburnt areas will be far less important in communities with a high proportion of individuals of species that resprout following a fire, such as fire‐adaptive savannas, grasslands and shrublands (Bond and Midgley 2001). In tropical fire‐sensitive ecosystems, the ability of protected areas to reduce fire risk is very variable, depending on the investment in protected area management (Laurance et al. 2012). In fire‐adaptive ecosystems, protected areas can effectively manage fires to enhance natural processes and biodiversity but may also overly suppress fires, thus leading to larger and more intensive wildfires that negatively impact biodiversity when they occur (Pivello 2011; Schmidt et al. 2018).

Fire type, whether prescribed or non‐prescribed, is also associated with how plants respond to and recover from fire, but it also depends on the ecosystem and taxa (Pastro et al. 2011; Rodrigues and Fidélis 2022). In fire‐sensitive ecosystems, especially tropical moist and dry broadleaf forests, where non‐prescribed fires are historically rare, they are increasing due to human‐induced land‐use change (Pivello 2011; Junior et al. 2018; Wimberly et al. 2024), coupled with severe drought events (Brando et al. 2014). Prescribed fire experiments in such ecosystems are rare and have not been reported in many scientific studies, and their effectiveness is poorly understood (Cochrane 2003; Brando et al. 2014; Roces‐Díaz et al. 2021). In fire‐adaptive ecosystems, where non‐prescribed fires are frequent, they are being reduced due to human influence on fire suppression and land‐management processes (Andela et al. 2017). In such ecosystems, prescribed burns are essential for managing fire and the ecosystem as they facilitate the increase of herbaceous species and reduce woody encroachment (Policelli et al. 2018). Moreover, biennial experimental fires promote forb species richness in tropical savannas (Rodrigues and Fidélis 2022).

Biodiversity gradually recovers following a fire event and should increasingly resemble the composition of the pre‐fire community as time increases, but it depends on the types of biomes and vegetation (Machida et al. 2021; Gorta et al. 2023). Frequent fire events can, however, prevent full recovery in closed ecosystems by driving fire‐sensitive species to regional extinction (Gallagher et al. 2021), and species recovery following a fire can be much slower in such biomes than in those that traditionally experience fire (Nelson et al. 2014).

In the current scenario of changing fire regimes driven by climate and land‐use change, understanding biodiversity conservation after a fire requires examining not just species counts but also the changes in community composition and species turnover (Gordijn et al. 2018; Durigan et al. 2020; Peterson and Reich 2008). Moreover, most studies assessing fire impacts on plant biodiversity focus on single study locations and fire characteristics. Meta‐analyses are scarce, but the relative fire sensitivity of native and exotic plant species has been assessed (Jauni et al. 2015; Alba et al. 2014; Aslan and Dickson 2020). Here, we build upon a systematic compilation of data from published studies of tropical and sub‐tropical plant community responses to fire. We assess post‐fire recovery of plant species richness and composition following fire events. Specifically, we test whether species richness and beta diversity (i.e., species turnover) between burnt and unburnt plots respond differently to time since fire and fire type (prescribed burns versus non‐prescribed burns). We also assess if protected area status (protected vs. unprotected) and biome types (fire sensitive vs. fire adaptive) moderate the responses of species richness and species turnover to fire events.

## 2 Methods

### 2.1 Literature Search

A systematic literature search was conducted following the PRISMA guidelines (Liberati et al. 2009; Moher et al. 2009) and completed in March 2023. Three searches were carried out using ‘Web of Science’, with the search terms: (i) fire* AND “species richness” AND plant*; (ii) burn* AND “species richness” AND plant*; and (iii) fire*AND “species richness” AND tree*. Our objective was to retain papers that were empirical field‐based studies conducted in the tropics or sub‐tropics, i.e., 30° north to 30° south (Corlett 2013), and that provided complete species lists for control (unburnt or sites sampled before a fire) and treatment sites (those with fires). We only selected studies with equal sampling effort between control and treatment sites since biases in study design can impact conclusions regarding fire impacts on biodiversity (Kelly et al. 2017).

The data collection process took place in five stages (Table 1). Following the systematic literature search, paper titles were scanned to identify studies relevant to understanding the impacts of fire on plant diversity in tropical and sub‐tropical locations. Next, duplicate papers were removed, and abstracts were read. Papers were only accepted if the study met our criteria of being an empirical field‐based study located in the tropics or sub‐tropics. We then read each paper in full and removed those for which sampling effort was uneven across control (unburnt) and treatment (burnt) sites or did not provide a complete species list. A list of retained papers is given in Appendix S1.

**TABLE 1:** The selection stages, procedure, and total number of papers obtained in the literature search.

Selection procedure	Number of papers
Papers yielded from initial search	8970
2 Papers left after scanning titles	1431
3 Papers left after removing duplicates	1065
4 Papers left after reading the abstract	460
5 Papers left after reading in full and checking selection criteria are met	28

### 2.2 Data Extraction & Quality Control

The final set of 28 studies contained 101 pairwise control (unburnt) and treatment (burnt) plots and 5311 observations, where one observation equates to a species being present in a burnt or unburnt plot (Appendix S2, Table S1). Some studies reported changes in tree and shrub communities but used plot sizes that are widely considered too small for accurate estimates of the species richness of these groups, as the plots could only contain one or two mature individuals of these life forms. Thus, we did not include ~3% of observations for calculating tree species richness when plots were less than 100 m2 or for shrub species richness when plots were less than 16 m^2^ (Mueller‐Dombois et al. 2008). We excluded these observations from both closed and open habitats to apply the same standard for all habitats. Moreover, we did not exclude all the sites or studies but only removed observations of tree/shrub species from the recordings in those smaller plots. Most studies (*n* = 24; 85%) provided their study site's latitude and longitude, but when these were not provided, they were obtained using the description of the study site location. Not all studies provided data on species' abundances (density or percentage cover), so we converted data into a presence/absence matrix for each burnt and unburnt site.

From each study, we extracted data on two fire metrics—time since fire (number of years between the most recent fire and sampling period) and fire type (either prescribed, including experimental and fire management burns, or non‐prescribed burns). Time since fire was converted to the finest temporal resolution possible; for example, if a study was conducted 4 months after the fire, we recorded this as 0.33 years (i.e., 4/12). We defined each site as protected if it was within the boundaries of a protected area (IUCN categories I to VI) as defined by the World Database on Protected Areas (WDPA) database (UNEP‐WCMC and IUCN 2020); this was achieved using the *wdpar* R package version 1.3.2 (Hanson 2020).

Biomes were first classified according to Olson et al. (2001), and then they were grouped into two levels: fire‐sensitive and fire‐adaptive, according to their vegetation type and historical exposure to fire. Tropical and Subtropical Moist Broadleaf Forests and Tropical and Subtropical Dry Broadleaf forests were grouped as fire sensitive and Tropical and Subtropical Coniferous Forests, Tropical and Subtropical Grasslands, Shrublands and Savannas and Flooded Grasslands and Savannas were grouped as fire‐adaptive biomes (Table 2).

**TABLE 2:** Classification of biomes into fire‐sensitive and fire‐adaptive according to the fire return interval.

Biome	Fire return interval	Category	References
Tropical and Subtropical Moist Broadleaf Forests	200–1000+ years (natural conditions); 5–10 years (with human influence)	Fire‐sensitive	Power et al. (2007) Brando et al. (2020) Cochrane (2003) Arrogante‐Funes et al. (2022)
Tropical and Subtropical Dry Broadleaf Forests	10–50 years	Fire‐sensitive	Arrogante‐Funes et al. (2022) Archibald et al. (2013)
Tropical and Subtropical Grasslands, Shrublands, and Savannas	1–5 years (frequent fires)	Fire‐adaptive	Bond and Keeley (2005); Staver et al. (2011) Arrogante‐Funes et al. (2022) Archibald et al. (2013)
Flooded Grasslands and Savannas	Variable (typically 1–10 years)	Fire‐adaptive	Damasceno‐Junior et al. (2021) Arrogante‐Funes et al. (2022) Archibald et al. (2013)
Tropical and Subtropical Coniferous Forests	3–16 years	Fire‐adaptive	Pausas (2015) Islas Madrid et al. (2013) Rodríguez‐Trejo and Fulé (2003).

### 2.3 Study Locations

**FIGURE 1:**
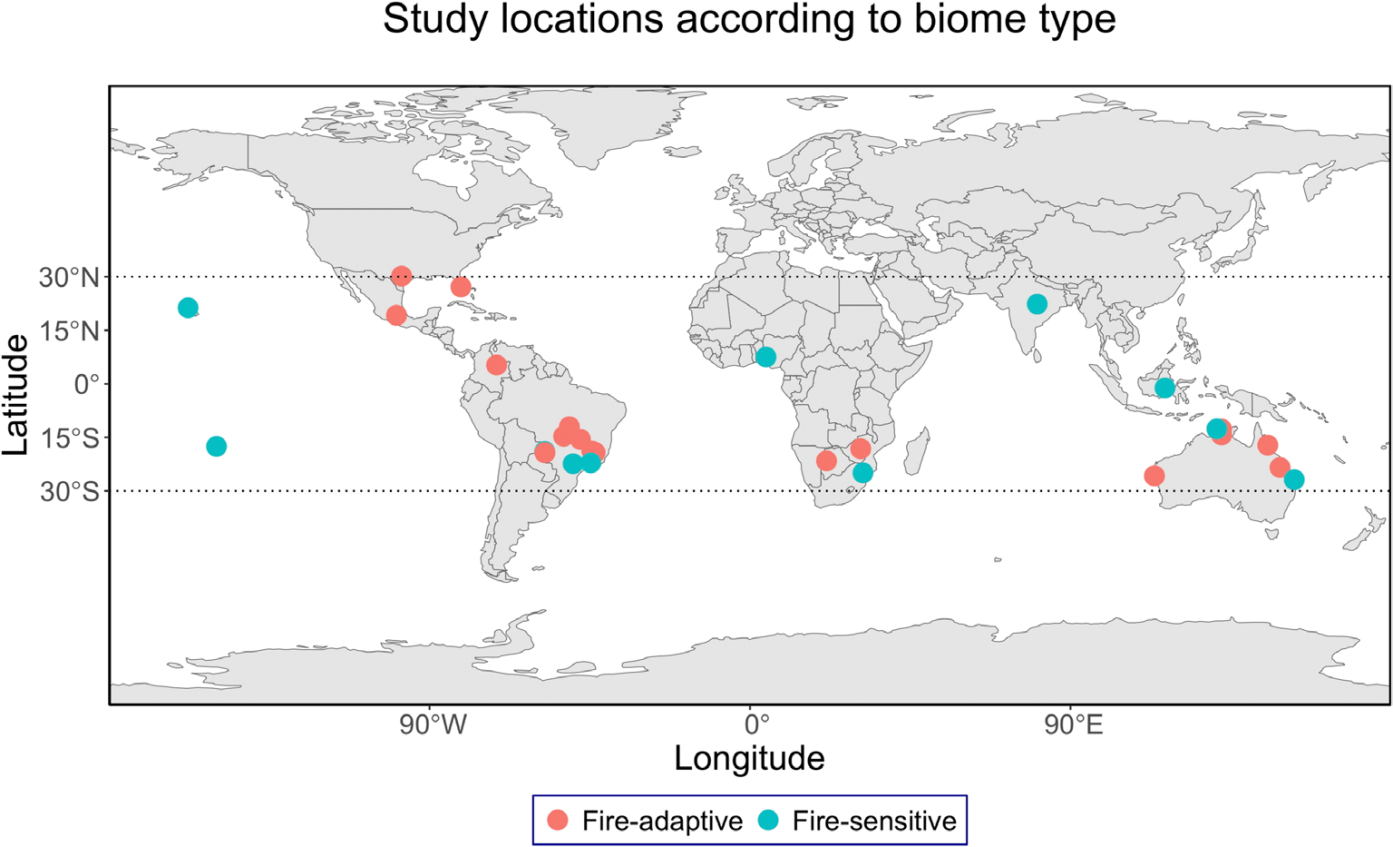
Study locations according to biome types (Fire‐adaptive, *n* = 17, Fire sensitive, *n* = 11). The dotted lines show the boundary of the sub‐tropical zone.

Our final 28 studies were located across the tropics (*n* = 22) and sub‐tropics (*n* = 6). However, studies from South America (*n* = 10) and Australasia (*n* = 7) dominated (Figure 1). Eleven studies were from the fire‐sensitive biomes (Tropical and Subtropical Moist Broadleaf Forests, Tropical and Subtropical Dry Broadleaf Forests), and 17 were from the fire‐adaptive biomes (Tropical and Subtropical Coniferous Forests, Tropical and Subtropical Grasslands, Shrublands and Savannas, and Flooded Grasslands and Savannas) (Figure 1).

### 2.4 Standardising Taxonomy and Life Form Classification

Species, genus, and family names were standardised according to The Plant List, R packages *Taxonstand* version 2.4 (Cayuela et al. 2012) and The World Flora, R package *WorldFlora* version 1.10 (Kindt 2020). Species that differ in their life‐history strategies, predominantly plants, can exhibit divergent recovery responses to fire (Maginel et al. 2019; Foster et al. 2018; Pilon et al. 2020; Chiminazzo et al. 2021). Hence, we classified each species into one of nine life forms: tree/shrub, forb, climber, graminoid, fern, succulent, lichen, and moss using eight datasets from the TRY database (Kattge et al. 2020); Botanical Information and Ecology Network (BIEN) database in R using the package *BIEN*, version 1.2.6 (Maitner et al. 2017) and AusTraits, a curated plant trait database for the Australian flora using the package *aurstraits* in R (Falster et al. 2021). This allowed us to classify 88% of species; the remaining species were classified using authenticated online sources or the life‐form classification used in the original study (Appendix S3, Table S2). Ferns, succulents, lichens, and mosses were excluded from further analysis as they were recorded in too few studies (≤ 5). We combined trees and shrubs into one category because we could not separate some species into trees and shrubs while standardising the life form groups. There were inconsistencies and overlaps in the classification of these two groups in our primary data sources, the TRY database, and other authenticated online sources. A list of plant groups and the number of (i) studies that recorded them, (ii) observations and (iii) sites recorded with protected and non‐protected sites in both fire‐sensitive and fire‐adaptive biome types are presented in Appendix S4, Table S3.

### 2.5 Biodiversity Metrics

We calculated two response variables (relative species richness—alpha diversity; beta‐diversity—pairwise dissimilarity) for each of the four analysed life forms, i.e., trees/shrubs, forbs, graminoids, and climbers. Relative species richness was calculated following Burivalova et al. (2014) as the total number of species in the burnt site divided by the total number of species in the unburnt site. Consequently, values of 1 represent no impact of fire on species richness, values of less than 1 denote reductions in species richness due to fire, while values greater than 1 signify increases in species richness.

Species turnover (beta diversity) was calculated as the Sørensen pairwise dissimilarity index (Sørensen 1948), which is widely used to measure the spatial turnover for presence/absence data in ecology and is independent of species richness (Koleff et al. 2003; Socolar et al. 2016). A value of 0 means the composition of two communities is identical, and a value of 1 means the two communities do not share any species in common.

### 2.6 Data Analysis

We modelled relative species richness and Sørensen index of (i) trees/shrubs, (ii) forbs, (iii) graminoids, and (iv) climbers using linear mixed‐effects methods with study ID as a random effect, using the lme4 package (Bates et al. 2015). We constructed all possible ecologically realistic models (*n* = 32; Appendix S5, Table S4) given our suite of predictor variables, that is., time since fire (years; ln transformed), fire type (fixed factor: prescribed/non‐prescribed burns), biome type (fixed factor: fire‐sensitive vs. fire‐adaptive), and protection status (fixed factor: protected/non‐protected). We included interaction terms between our two fire metrics (time since fire and fire type) and (i) biome type and (ii) protection status to test whether biome type or protected area status moderated the relationships between each fire metric and our outcome variables.

We used D^2^ as a measure of explanatory capacity; *D* ^2^ = (ND − RD)/ND where ND is the null deviance and RD is the residual deviance, which cannot be explained by the model; thus, ‘ND–RD’ is the explained deviance. *D* ^2^ varies between zero and one and equals one when the deviance can be explained completely by the model (Guisan and Zimmermann 2000).

We used an information‐theoretic criterion approach to obtain a set of models whose Δ AICc values were within two points of the best‐performing model, that is, that with the lowest AICc value, and then conducted model averaging (Burnham and Anderson 2004).

All analyses were conducted in R 4.4.2 (R Core Development Team 2024). Continuous variables were centred prior to analysis, and we used the equivalent sum to zero contrasts approach for categorical variables (Schielzeth 2010). Centering variables reduces problems that otherwise arise with model averaging when interaction terms are included as predictors (Schielzeth 2010; Cade 2015; Tyre 2017).

In all cases, models had Variance Inflation Factors (VIF) < 10, indicating that results are not markedly impacted by collinearity between predictors (Hair jr. et al. 1992; Craney and Surles 2002). We also checked for the linearity of responses by including square terms and comparing the model fit to equivalent models that only included a linear term. The fit of all models was also checked using model diagnostic plots.

## 3 Results

### 3.1 Relative Species Richness

Model averaging of the relative species richness models shows the effect of fire on the relative species richness of trees/shrubs and forbs only. We did not observe the impacts of fire on the relative species richness of forbs and graminoids because the null model (i.e., one that lacked predictors) was found to be the best model for forbs and graminoid relative species richness.

**FIGURE 2:**
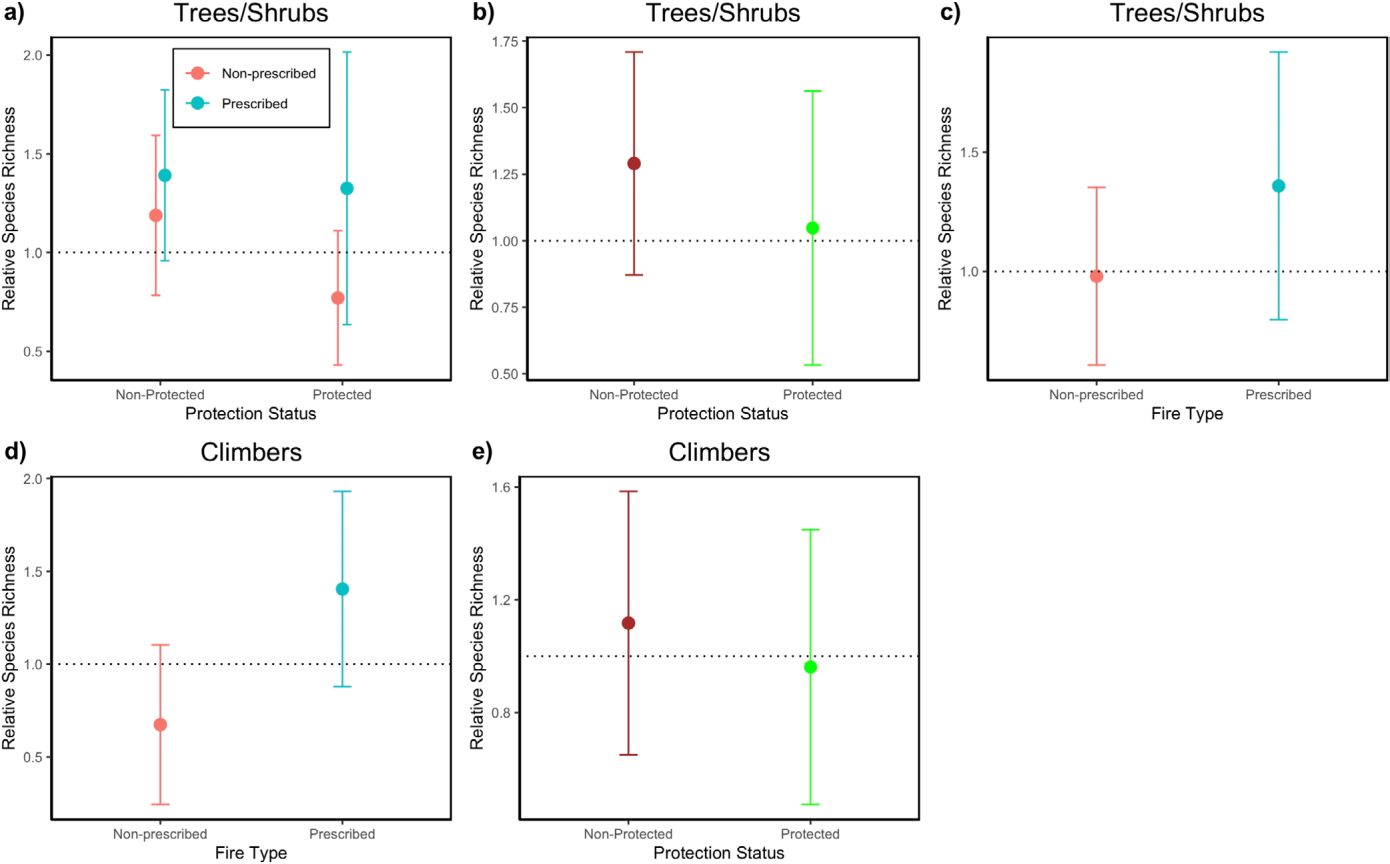
Impact of fire on relative species richness species richness in burnt sites divided by richness in the control sites (a, b & c) Trees/Shrubs: Non‐protected (*n* = 38), protected (*n* = 46); Non‐prescribed (*n* = 59), prescribed (*n* = 25) and (d & e) Climbers Non‐prescribed (*n* = 33), Prescribed (*n* = 27), Non‐protected (*n* = 35), protected (*n* = 25). The error bars represent the 95% confidence interval. The dotted lines represent a relative species richness of 1, that is., equal species richness in both burnt and unburnt plots.

Models of the relative species richness of trees/shrubs in burnt and unburnt plots had extremely low explanatory power (i.e., 3.98%), and model averaging of best models revealed protection status, fire type, and the interaction between fire type and protection status influenced the relative species richness (Table 2). However, the 95% confidence interval of the parameter estimates of all predictors in the model averaging overlapped zero. The interaction between protection status and fire type revealed that in non‐protected sites, relative species richness increased in plots experiencing both prescribed and non‐prescribed burns relative to unburnt controls (Table 3; Figure 2a). However, in protected sites, prescribed burns increased species richness in burnt relative to unburnt controls, whereas non‐prescribed burns resulted in decreased species richness in burnt relative to unburnt controls (Table 3; Figure 2a). In the case of protection status only, relative species richness increased at burnt sites relative to unburnt controls in unprotected sites, whilst within protected sites, species richness was more similar in burnt and unburnt sites (Table 3; Figure 2b). In the case of the fire type, species richness increased at burnt sites relative to unburnt controls in prescribed burn sites, whilst within the non‐prescribed sites, species richness was more similar in burnt and unburnt sites (Table 2, Figure 2c).

**TABLE 3:** Results from model averaging across multiple regression models of relative species richness in burnt sites relative to control (unburnt) sites for trees/shrubs and climbers. Parameter estimates are provided with 95% confidence intervals in brackets.

Predictors	Life Forms	
	Trees/shrubs	Climbers
	Parameter estimate (95% CI)	
Fire Type (Non‐prescribed)	−0.189 (−0.451, 0.072)	−0.365* (−0.672, −0.057)
Protection Status (Non‐protected)	0.121 (−0.118, 0.360)	0.077 (−0.188, 0.344)
Fire Type (Non‐prescribed): Protection Status (Non‐protected)	0.088 (−0.154, 0.330)	
Model Explanatory Power (*D* ^2^)	3.98%	5.17%

* Shows where the predictor has a significant relationship with the response variable (i.e., relative species richness).

Models of the relative species richness of climbers in burnt and unburnt sites also had very low explanatory power (i.e., 5.17%). Model averaging revealed that prescribed burns increased climber richness in burnt plots relative to unburnt ones, whilst non‐prescribed burns decreased climber richness in burnt sites relative to unburnt controls (Table 3; Figure 2d). Similarly, climber richness was slightly higher in burnt plots relative to unburnt in non‐protected sites, whereas it remained similar in burnt plots relative to unburnt in protected sites, but the 95% confidence interval overlapped zero (Table 3, Figure 2e).

### 3.2 Species Turnover

Models of turnover in species composition of tree/shrub, forb, graminoid and climber communities between burnt and unburnt plots consistently had good explanatory power (trees/shrubs: 51.02%, forbs: 27.60%, graminoids: 64.95%, and climbers 17.48%). Model averaging of the species turnover of all four life form groups also showed variation in the impact of fire across these groups. Trees/shrubs had more than one best model within 2 ∆AICc, whereas forbs, graminoids, and climbers had only one best model within 2 ∆AICc.

**FIGURE 3:**
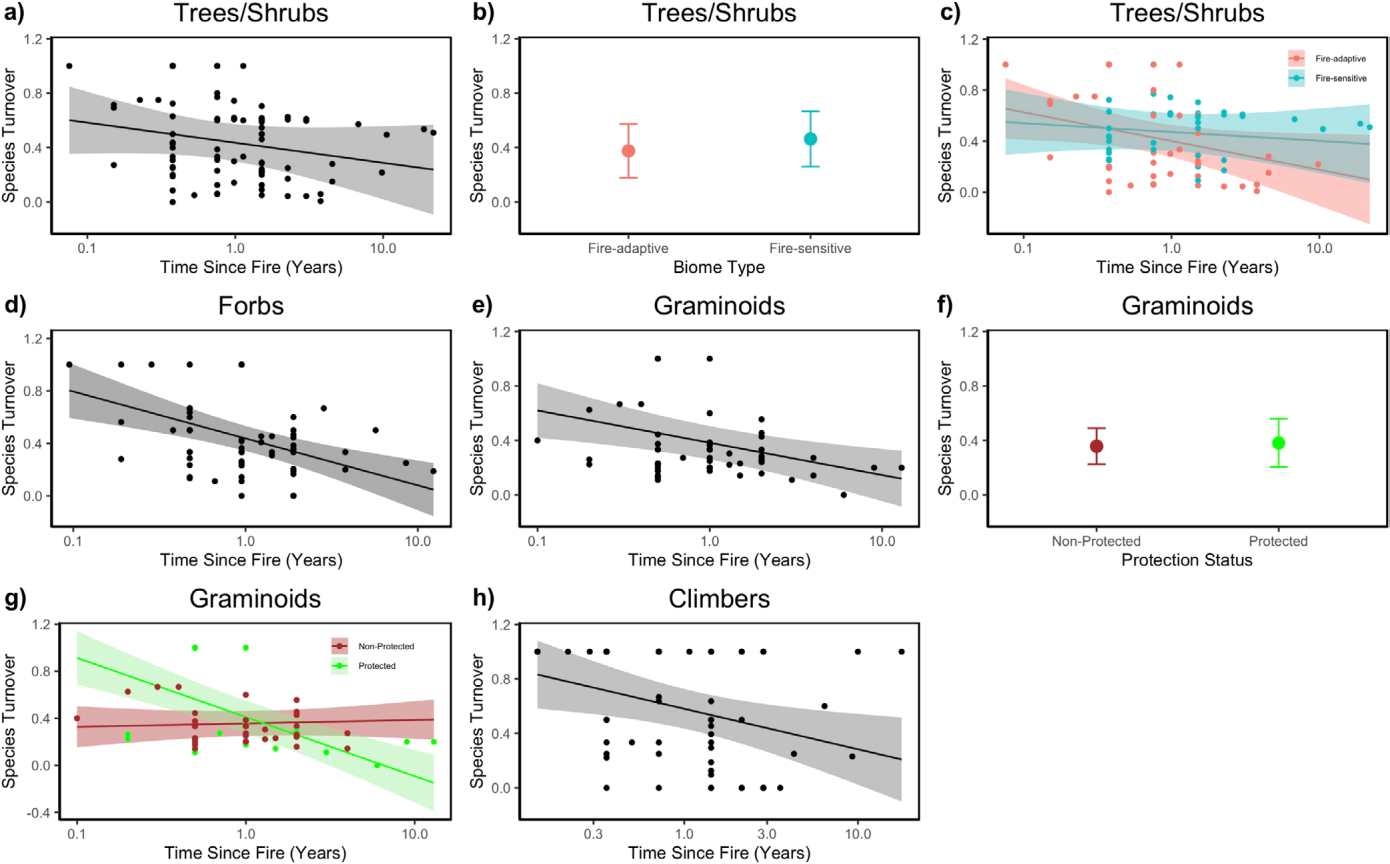
Impact of fire on species turnover between burnt and unburnt sites on (a, b & c) Trees/Shrubs, (*n* = 84, fire‐adaptive = 43, fire‐sensitive = 41), (d) Forbs (*n* = 61); (e, f, g) Graminoids: (*n* = 56, non‐protected = 40, protected =16), and Climbers: Fire adaptive (*n* = 60). Each point represents the no. of sites. The *x*‐axis for (a, c, d, e, g & h) (i.e., time since fire) is plotted on a log scale. The shaded area for each plot represents the 95% confidence interval.

The model averaging of the best models of trees/shrubs showed that time as fire, biome type, and the interaction between time since fire and biome type influenced species turnover. We observed that the time as fire had a negative effect on species turnover. The dissimilarity of species between burnt and unburnt plots was initially marked at ~0.6 and decreased gradually, and about 25% of the reduction in the dissimilarity of species was observed in ~5 years and about 50% within ~10 years (Table 4, Figure 3a). Although the biome type and the interaction between time since fire and biome type also influenced the species turnover of trees/shrubs, the 95% confidence interval of parameter estimates overlapped zero (Table 4). We observed a higher species turnover in fire‐sensitive biomes than in fire‐adaptive biomes (Table 4, Figure 3c). Species turnover was reduced gradually in fire‐adaptive biomes but progressively increased in fire‐sensitive biomes (Table 4, Figure 3b).

**TABLE 4:** Results from model averaging across multiple regression models of species turnover for trees/shrubs, forbs, graminoids and climbers. Parameter estimates are provided with 95% confidence intervals in brackets.

Predictors	Life Forms			
	Trees/shrubs	Forbs	Graminoids	Climbers
	Parameter estimate (95% CI)			
Time Since Fire (ln transformed)	−0.063* (−0.122, −0.006)	−0.155* (−0.235, −0.076)	−0.102* (−0.160, −0.044)	−0.129* (−0.231, −0.027)
Protection Status (Non‐protected)			−0.027 (−0.141, 0.084)	
Time Since Fire (ln): Protection Status (Non‐Protected)			0.115* (0.051, 0.176)	
Biome Type (Fire‐adaptive)	−0.035 (−0.131, 0.060)			
Time Since Fire (ln): Biome Type (Fire‐adaptive)	−0.034 (−0.103, 0.035)			
Model Explanatory Power (D^2^)	49.48%	27.60%	64.95%	17.48%

* Shows where the predictor has a significant relationship with the response variable (i.e., relative species richness).

The best model of forbs revealed that only the predictor time since fire influenced species turnover. The turnover of forb communities was ~0.8 immediately after the fire but had reduced to ~0.4 (50%) within a year and about ~0.2 (75%) in 5 years (Table 4, Figure 3d).

Similarly, the best model of Graminoids showed that time since fire, protection status, and the interaction between time since fire and protection influenced the turnover of species composition (Table 4). Time since fire indicated that immediately after the fire, the dissimilarity was (~6) between burnt and unburnt plots but reduced to 50% nearly within ~2 years. Regarding the protection status, we observed a similar level of species turnover at protected and non‐protected sites. The interaction between time since fire and protection status revealed the opposite trend in protected and non‐protected sites. Immediately after the fire, the protected area experienced higher turnover than the non‐protected areas. Thereafter, the dissimilarity decreased sharply in protected sites as time progressed and became identical in both burned and unburnt plots for about ~5 years. In contrast, the dissimilarity increased slowly but remained similar in burnt and unburnt plots in non‐protected sites.

There was only one best model in climbers within 2 ∆AICc. The best model showed that the only predictor, time since fire, influenced species turnover between burnt versus unburnt plots. Climber turnover was (~8) immediately after the fire, but reduced to (~0.5, i.e., 50%) in ~3 years and gradually reduced.

## 4 Discussion

Fire has played an important role in shaping tropical biodiversity for millennia; it is essential for maintaining biodiversity in fire‐adaptive ecosystems but harmful to the biodiversity of fire‐sensitive ecosystems (He et al. 2019; Kelly et al. 2020). However, in recent decades, climate and land use changes have affected the frequency and pattern of fires in both fire‐sensitive and fire‐adaptive ecosystems, leading to changes in vegetation structure and biodiversity (Kelly et al. 2020; Cochrane and Barber 2009; He et al. 2019). This creates uncertainty regarding how future changes in fire regimes will influence plant biodiversity (Kelly et al. 2020; McLauchlan et al. 2020). To investigate this uncertainty, we analysed a systematic compilation of data quantifying species richness and community composition responses to fire in tropical communities of major plant life forms. Our analyses account for quantifying plant responses to time since fire and fire type (prescribed or non‐prescribed burns) and assess if protected area status and biome types (fire sensitive vs. fire adaptive) modify plant community responses.

Despite conducting a comprehensive literature search, we only found 28 studies that met our data analysis requirements. Thus, additional empirical fieldwork is needed to assess plant community responses to fire; such studies should follow the open science principles of making underlying datasets freely available to facilitate meta‐analyses. Our focal studies included ones that assessed biodiversity recovery up to 29 years following fire events, but most studies were conducted within 10 years of a fire event. Given that we find plant community composition can remain impacted by fires 10 years after they occur (see below discussion), there is a particular need for longitudinal studies in the long term (> 10 years). Our results indicate that changes in species richness and recovery of community composition following fire events vary across plant life forms. We thus encourage future studies to consider the variation across life forms' responses to fire in their study design and interpretation. We observed that ecosystems, such as flooded savannas and coniferous forests, are poorly represented within our dataset, further underlining the need for additional field studies in these ecosystems.

Fire impact can vary depending on fire‐associated variables such as fire frequency (Pati et al. 2024) and anthropogenic disturbance variables such as grazing, logging, mining, etc. (Kelly and Brotons 2017). Although these variables are important determinants of the impact of fire on plant diversity and post‐fire recovery, we have not been able to consider this because all the studies that met our criteria did not consistently report additional metrics of fire history. Future studies of fire impacts on biodiversity should consistently report the site's full fire history, including data on the fire season and fire return interval and specific anthropogenic pressures impacting the study area.

Due to the lack of a precise classification of trees and shrubs in our focal studies and TRY database, we had to combine trees and shrubs into a single category. Although it has been common practice to report the impact of fire on woody vegetation, that is trees and shrubs together (e.g., Nóbrega et al. 2019; Smit et al. 2010), it is important to note that the findings could have been different if trees and shrubs were analysed separately as they can have different strategies for responding to the loss of above‐ground biomass in fires (Chiminazzo et al. 2021, 2023). Future studies should explicitly classify trees and shrubs into distinct categories where possible. Despite data availability limitations, our analyses provide important novel preliminary insights regarding biodiversity responses and recovery from fire events. The results from the relative species richness models should be taken very cautiously, as these models have extremely limited explanatory power. This could be due to the unavailability of long‐term data and the shortage of some important fire‐related variables, such as fire return interval (frequency), season, duration, etc., in the primary studies.

### 4.1 Variation Across Biomes

Our analyses reveal very weak evidence about the divergent responses of trees/shrub species turnover to fire events depending on the biome type in which they are located. These relationships are further associated with time since fire. Our results indicate that the tree/shrub species turnover remained higher in fire‐sensitive biomes than in fire‐adaptive biomes. Species turnover is gradually reduced in the fire‐adaptive biomes, with a ~50% reduction in dissimilarity within ~5 years, whereas the dissimilarity remains unchanged in the fire‐sensitive biomes. This could be because woody species in fire‐adapted biomes are regularly exposed to fire, possess fire‐resistant traits, and can regenerate and recover quickly in fire fire‐adapted biomes (Brando et al. 2011; Keeley et al. 2011). Our findings should be approached cautiously, considering the limited explanatory power and significance levels of the employed models and because some previous studies (e.g., Feng et al. 2021; Kodandapani et al. 2004; Andersen et al. 2005; et al., 2019) suggest that the increasing frequency of fire events across the tropics can negatively impact trees regardless of biome types.

### 4.2 Protection Status

Species richness of trees/shrubs and climbers increased following fires in non‐protected sites but tended to remain similar in protected sites. The species richness of trees/shrubs and protection status is further influenced by fire type (prescribed vs. non‐prescribed). The species richness in protected areas decreased compared to non‐protected areas in both trees/shrubs and climbers. Fire suppression in protected areas can result in a substantial accumulation of flammable material that increases the adverse ecological consequences of fires when they arise and encourages the formation of assemblies that are more sensitive to fire than areas lacking protection (De Groot et al. 2009; Pereira et al. 2012; Pivello 2011). Similarly, the species turnover of graminoid community composition remained similar in both protected and non‐protected sites. However, the reduction in species turnover is sharp in protected sites compared to non‐protected sites. Graminoid species are fire‐adaptive, regardless of protection, and should recover to similar levels, but the reasons for these differences remain unclear. The persistence of higher species turnover in unprotected areas could be the result of the arrival of non‐grass species due to the higher level of disturbance in the post‐fire recovery process. Another possible reason for this could be that protected areas experience lower anthropogenic pressure compared to non‐protected areas (Geldmann et al. 2019; Andam et al. 2008) and facilitate suitable ecological conditions for species recovery and regeneration (Gray et al. 2016), supporting the stabilising influence of protected areas.

### 4.3 Effects of Fire Type

We found an extremely weak signal that the impact of fire type varies in trees/shrubs and climbers' richness. Both the trees/shrubs and climber's species richness increased in prescribed burn sites compared to the non‐prescribed. Prescribed burning can increase seed germination rates and lower sapling mortality, leading to greater species richness (López‐Cruz et al. 2022). These results extend previous work suggesting reduced species richness of climbers in burnt compared to unburnt plots, irrespective of fire type (e.g., Addo‐Fordjour et al. 2020; Balch et al. 2011). In the case of trees/shrubs, the impact of fire type was also moderated by protection status (discussed in the protection status section). Although we observed some trends in the effects of fire type on the trees/shrubs and climbers models, these results should be interpreted cautiously due to the extremely low explanatory power and the low significance level of the best models.

### 4.4 Effects of Time Since Fire

Whilst we find no effects of time since fire on species richness, the species composition of tree/shrub, forbs, graminoid and climber communities changes markedly following a fire. The result shows great variation in the recovery of species composition across our focal life form groups. We observed that over the period, the species dissimilarity of forbs decreased faster than all other life form groups. Nearly 50% of the recovery of species composition of the forb's community was observed within 1 year, followed by climbers in 3 years, graminoids in 4 and trees/shrubs in 10 years. A longer period since the last burn positively impacts plant species recovery as they can regrow and fully mature, assuming they are not ruderal species dependent on the open conditions generated immediately after a fire (Plumanns‐Pouton et al. 2023; Morrison et al. 1995; Arroyo‐Vargas et al. 2019). However, the invasion of alien species has become a major concern as they outcompete native plants, making it difficult for species composition to recover at their optimum level after a fire (Alba et al. 2014; Jauni et al. 2015). We further observed that the recovery of graminoid species varies across protection status.

As mentioned in the protection status section, recovery was faster in protected compared to unprotected graminoid. The long‐term persistence of these compositional changes is probably driven by multiple factors, including the long‐term legacy of altered nutrient availability post‐fire (Verma et al. 2019), fire‐induced reductions in tree growth rates (Bucini and Hanan 2007), and (especially outside protected areas) altered land‐use patterns following fire events (Butsic et al. 2015). The recovery rate of tree/shrub species composition is slower in fire‐sensitive biomes than in fire‐adaptive biomes. About 50% of species recovery is observed in approximately 5 years in fire‐adaptive biomes, whereas only 25% is observed in fire‐sensitive biomes over the same period. It is notable that fire return rates in many tropical forests, especially fire‐sensitive forests, are getting shorter in recent decades (Archibald et al. 2013), and in some locations, such as the central highlands of Vietnam, have increased in recent decades primarily due to changes in human activity (Nguyen et al. 2023). Indeed, the number of fires in tropical forests (both dry and humid) has increased at ~5% per annum since 2001 (Tyukavina et al. 2022) and is projected to increase further due to both climate change and human activity across a wide range of tropical ecosystems (Wu et al. 2021; Li et al. 2023). Thus, increasing fire frequency in the tropics may change the recovery rate of species composition of all life forms, and such impact not only depends on the time since fire but also on the protection status and biome types.

## 5 Conclusions

Our data compilation and analysis of tropical/sub‐tropical plant community responses to fire generate important findings that inform knowledge of fire impacts and mitigation strategies and help shape future research agendas. Despite increasing awareness of changing tropical fire regimes, limited studies address plant community responses to key fire features, and long‐term longitudinal studies that can quantify recovery times are particularly scarce. More focused research is needed to assess how species recovery rates are influenced by landscape composition and configuration. We uncover considerable heterogeneity across plant life forms in their responses to fire metrics and encourage researchers to consider this when reporting fire impact studies.

Our research makes some important contributions. We uncover evidence that fire impacts on species richness and recovery of community composition can vary with protection status, with protected areas appearing to be able to support graminoid species composition from fire‐induced changes. Similarly, we recognised that prescribed burns can enhance the species richness of trees/shrubs and climbers compared to non‐prescribed burns. In addition, there were differences in fire impacts between fire‐adapted and fire‐sensitive biomes regarding the dissimilarity in community composition of trees/shrubs. However, this relationship should be interpreted carefully due to the models' extremely low significance level and explanatory power. We also observe changes in the species composition of all plant growth forms, with fire effects influenced by protection status and biome type. Tropical and subtropical plant communities may undergo compositional changes due to observed and projected future increases in fire frequency, which could shorten species recovery time between fire events.

## Author Contributions

**Dharma P. Sapkota:** conceptualization (equal), data curation (lead), formal analysis (lead), methodology (equal), visualization (lead), writing – original draft (lead). **Karl L. Evans:** conceptualization (equal), data curation (supporting), formal analysis (supporting), methodology (equal), supervision (lead), visualization (supporting), writing – original draft (supporting), writing – review and editing (equal). **David P. Edwards:** conceptualization (equal), data curation (supporting), formal analysis (supporting), methodology (equal), supervision (supporting), visualization (supporting), writing – original draft (supporting), writing – review and editing (equal). **Mike R. Massam:** formal analysis (supporting), methodology (supporting), visualization (supporting), writing – review and editing (supporting).

## Conflicts of Interest

The authors declare no conflicts of interest.

## Supporting information


Appendix S1.

Appendix S2.

Appendix S3.

Appendix S4.

Appendix S5.

## Data Availability

All data included in analyses are available in the Supporting Information.

## References

[ece371018-bib-0001] Abreu, R. C. R. , W. A. Hoffmann , H. L. Vasconcelos , N. A. Pilon , D. R. Rossatto , and G. Durigan . 2017. “The Biodiversity Cost of Carbon Sequestration in Tropical Savanna.” Science Advances 3, no. 8: e1701284. 10.1126/sciadv.1701284. 28875172 PMC5576881

[ece371018-bib-0002] Addo‐Fordjour, P. , F. Kadan , Z. B. Rahmad , D. Fosu , and B. Ofosu‐Bamfo . 2020. “Wildfires Cause Shifts in Liana Community Structure and Liana‐Soil Relationships in a Moist Semi‐Deciduous Forest in Ghana.” Folia Geobotanica 55, no. 4: 273–287. 10.1007/s12224-020-09380-6.

[ece371018-bib-0003] Aide, T. M. , M. L. Clark , H. R. Grau , et al. 2012. “Deforestation and Reforestation of Latin America and the Caribbean (2001‐2010).” Biotropica 45, no. 2: 262–271. 10.1111/j.1744-7429.2012.00908.x.

[ece371018-bib-0004] Alba, C. , H. Skálová , K. F. McGregor , C. D'Antonio , and P. Pyšek . 2014. “Native and Exotic Plant Species Respond Differently to Wildfire and Prescribed Fire as Revealed by Meta‐Analysis.” Journal of Vegetation Science 26, no. 1: 102–113. 10.1111/jvs.12212.

[ece371018-bib-0005] Alroy, J. 2017. “Effects of Habitat Disturbance on Tropical Forest Biodiversity.” Proceedings of the National Academy of Sciences 114, no. 23: 6056–6061. 10.1073/pnas.1611855114. PMC5468684 28461482

[ece371018-bib-0006] Andam, K. S. , P. J. Ferraro , A. Pfaff , G. A. Sanchez‐Azofeifa , and J. A. Robalino . 2008. “Measuring the Effectiveness of Protected Area Networks in Reducing Deforestation.” Proceedings of the National Academy of Sciences 105, no. 42: 16089–16094. 10.1073/pnas.0800437105. PMC2567237 18854414

[ece371018-bib-0007] Andela, N. , D. C. Morton , L. Giglio , et al. 2017. “A Human‐Driven Decline in Global Burned Area.” Science 356, no. 6345: 1356–1362. 10.1126/science.aal4108. 28663495 PMC6047075

[ece371018-bib-0008] Andersen, A. N. , G. D. Cook , L. K. Corbett , et al. 2005. “Fire Frequency and Biodiversity Conservation in Australian Tropical Savannas: Implications From the Kapalga Fire Experiment.” Austral Ecology 30, no. 2: 155–167. 10.1111/j.1442-9993.2005.01441.x.

[ece371018-bib-0009] Aragão, L. E. O. C. , Y. Malhi , N. Barbier , et al. 2008. “Interactions Between Rainfall, Deforestation and Fires During Recent Years in the Brazilian Amazonia.” Philosophical Transactions of the Royal Society, B: Biological Sciences 363, no. 1498: 1779–1785. 10.1098/rstb.2007.0026. PMC2373892 18267907

[ece371018-bib-0010] Archibald, S. , C. E. R. Lehmann , J. L. Gomez‐Dans , and R. A. Bradstock . 2013. “Defining Pyromes and Global Syndromes of Fire Regimes.” Proceedings of the National Academy of Sciences 110, no. 16: 6442–6447. 10.1073/pnas.1211466110. PMC3631631 23559374

[ece371018-bib-0011] Arrogante‐Funes, F. , I. Aguado , and E. Chuvieco . 2022. “Global Assessment and Mapping of Ecological Vulnerability to Wildfires.” Natural Hazards and Earth System Sciences 22, no. 9: 2981–3003. 10.5194/nhess-22-2981-2022.

[ece371018-bib-0012] Arroyo‐Vargas, P. , A. Fuentes‐Ramírez , B. Muys , and A. Pauchard . 2019. “Impacts of Fire Severity and Cattle Grazing on Early Plant Dynamics in Old‐Growth Araucaria‐Nothofagus Forests.” Forest Ecosystems 6, no. 1:1–14. 10.1186/s40663-019-0202-2.

[ece371018-bib-0013] Aslan, C. , and B. Dickson . 2020. “Non‐native Plants Exert Strong but Under‐Studied Influence on Fire Dynamics.” NeoBiota 61: 47–64. 10.3897/neobiota.61.51141.

[ece371018-bib-0014] Balch, J. K. , D. C. Nepstad , L. M. Curran , et al. 2011. “Size, Species, and Fire Behavior Predict Tree and Liana Mortality From Experimental Burns in the Brazilian Amazon.” Forest Ecology and Management 261, no. 1: 68–77. 10.1016/j.foreco.2010.09.029.

[ece371018-bib-0015] Barlow, J. , E. Berenguer , R. Carmenta , and F. França . 2019. “Clarifying Amazonia's Burning Crisis.” Global Change Biology 26: 319–321. 10.1111/gcb.14872. 31729092

[ece371018-bib-0016] Bates, D. , M. Mächler , B. Bolker , and S. Walker . 2015. “Fitting Linear Mixed‐Effects Models Using lme4.” Journal of Statistical Software 67, no. 1: 1–48. 10.18637/jss.v067.i01.

[ece371018-bib-0017] Bond, W. , and J. Keeley . 2005. “Fire as a Global ‘Herbivore’: The Ecology and Evolution of Flammable Ecosystems.” Trends in Ecology & Evolution 20, no. 7: 387–394. 10.1016/j.tree.2005.04.025. 16701401

[ece371018-bib-0019] Bond, W. J. , and J. J. Midgley . 2001. “Ecology of Sprouting in Woody Plants: The Persistence Niche.” Trends in Ecology & Evolution 16, no. 1: 45–51. 10.1016/s0169-5347(00)02033-4. 11146144

[ece371018-bib-0020] Brando, P. , M. Macedo , D. Silvério , et al. 2020. “Amazon Wildfires: Scenes From a Foreseeable Disaster.” Flora 268: 151609. 10.1016/j.flora.2020.151609.

[ece371018-bib-0021] Brando, P. M. , J. K. Balch , D. C. Nepstad , et al. 2014. “Abrupt Increases in Amazonian Tree Mortality due to Drought–Fire Interactions.” Proceedings of the National Academy of Sciences 111, no. 17: 6347–6352. 10.1073/pnas.1305499111. PMC4035969 24733937

[ece371018-bib-0022] Brando, P. M. , D. C. Nepstad , J. K. Balch , et al. 2011. “Fire‐Induced Tree Mortality in a Neotropical Forest: The Roles of Bark Traits, Tree Size, Wood Density and Fire Behavior.” Global Change Biology 18, no. 2: 630–641. 10.1111/j.1365-2486.2011.02533.x.

[ece371018-bib-0023] Bucini, G. , and N. P. Hanan . 2007. “A Continental‐Scale Analysis of Tree Cover in African Savannas.” Global Ecology and Biogeography 16: 593–605. 10.1111/j.1466-8238.2007.00325.x.

[ece371018-bib-0024] Burivalova, Z. , Ç. Şekercioğlu , and L. Koh . 2014. “Thresholds of Logging Intensity to Maintain Tropical Forest Biodiversity.” Current Biology 24, no. 16: 1893–1898. 10.1016/j.cub.2014.06.065. 25088557

[ece371018-bib-0025] Burnham, K. P. , and D. R. Anderson . 2004. “Multimodel Inference.” Sociological Methods & Research 33, no. 2: 261–304. 10.1177/0049124104268644.

[ece371018-bib-0026] Busch, J. , and K. Ferretti‐Gallon . 2017. “What Drives Deforestation and What Stops It? A Meta‐Analysis.” Review of Environmental Economics and Policy 11, no. 1: 3–23. 10.1093/reep/rew013.

[ece371018-bib-0027] Butsic, V. , M. Kelly , and M. Moritz . 2015. “Land Use and Wildfire: A Review of Local Interactions and Teleconnections.” Landscape 4, no. 1: 140–156. 10.3390/land4010140.

[ece371018-bib-0028] Cade, B. S. 2015. “Model Averaging and Muddled Multimodal Inferences.” Ecology 96: 2370–2382. 10.1890/14-1639.1. 26594695

[ece371018-bib-0029] Cayuela, L. , I. Granzow‐de la Cerda , F. S. Albuquerque , and J. D. Golicher . 2012. “Taxonstand: An R Package for Species Names Standardisation in Vegetation Databases.” Methods in Ecology and Evolution 3, no. 6: 1078–1083. 10.1111/j.2041-210X.2012.00232.x.

[ece371018-bib-0030] Charles‐Dominique, T. , A. C. Staver , G. F. Midgley , and W. J. Bond . 2015. “Functional Differentiation of Biomes in an African Savanna/Forest Mosaic.” South African Journal of Botany 101: 82–90. 10.1016/j.sajb.2015.05.005.

[ece371018-bib-0031] Chiminazzo, M. A. , A. B. Bombo , T. Charles‐Dominique , and A. Fidelis . 2021. “Your Best Buds Are Worth Protecting: Variation in Bud Protection in a Fire‐Prone Cerrado System.” Functional Ecology 35: 2424–2434. 10.1111/1365-2435.13907.

[ece371018-bib-0032] Chiminazzo, M. A. , T. Charles‐Dominique , D. R. Rossatto , A. B. Bombo , and A. Fidelis . 2023. “Why Woody Plant Modularity Through Time and Space Must Be Integrated in Fire Research?” AoB Plants 15, no. 3: plad029. 10.1093/aobpla/plad029. 37288427 PMC10243913

[ece371018-bib-0033] Cochrane, M. A. 2003. “Center for Global Change and Earth Observations, and Instituto do Homeme Meio Ambiente da Amazônia (IMAZON) Fire Science for Rainforests.” Nature 421: 913. https://www.camafu.org.mx/wp‐content/uploads/2017/12/Fire_Science_for_Rainforest.pdf. 12606992 10.1038/nature01437

[ece371018-bib-0034] Cochrane, M. A. , and C. Barber . 2009. “Climate Change, Human Land Use and Future Fires in the Amazon.” Global Change Biology 15, no. 3: 601–612. 10.1111/j.1365-2486.2008.01786.x.

[ece371018-bib-0035] Cochrane, M. A. , and M. D. Schulze . 1999. “Fire as a Recurrent Event in Tropical Forests of the Eastern Amazon: Effects on Forest Structure, Biomass, and Species Composition.” Biotropica 31: 2–16. 10.1111/j.1744-7429.1999.tb00112.x.

[ece371018-bib-0037] Corlett, R. T. 2013. “Where Are the Subtropics?” Biotropica 45: 273–275. 10.1111/btp.12028.

[ece371018-bib-0038] Craney, T. A. , and J. G. Surles . 2002. “Model‐Dependent Variance Inflation Factor Cutoff Values.” Quality Engineering 14, no. 3: 391–403. 10.1081/qen-120001878.

[ece371018-bib-0039] Damasceno‐Junior, G. A. , J. O. Alexandre , P. Parolin , and A. Pott . 2021. “Fire, Flood and Pantanal Vegetation.” Plant and Vegetation: 18: 661–688. 10.1007/978-3-030-83375-6_18.

[ece371018-bib-0040] Dantas, V. , M. Hirota , R. S. Oliveira , and J. G. Pausas . 2016. “Disturbance Maintains Alternative Biome States.” Ecology Letters 19, no. 1: 12–19. 10.1111/ele.12537. 26493189

[ece371018-bib-0041] De Groot, W. J. , M. D. Flannign , and A. Cantin . 2009. “Adapting Fire Management to Future Fire Regimens: Impact on Boreal Forest Composition and Carbon Balance in Canadian National Parks.” Geophysical Research Abstracts 11: 13659.

[ece371018-bib-0042] Driscoll, D. A. , D. B. Lindenmayer , A. F. Bennett , et al. 2010. “Fire Management for Biodiversity Conservation: Key Research Questions and Our Capacity to Answer Them.” Biological Conservation 143, no. 9: 1928–1939. 10.1016/j.biocon.2010.05.026.

[ece371018-bib-0043] Durigan, G. , N. A. L. Pilon , R. C. R. Abreu , et al. 2020. “No Net Loss of Species Diversity After Prescribed Fires in the Brazilian Savanna.” Frontiers in Forests and Global Change 19: 13. 10.3389/ffgc.2020.00013.

[ece371018-bib-0044] Edwards, D. P. , J. B. Socolar , S. C. Mills , Z. Burivalova , L. P. Koh , and D. S. Wilcove . 2019. “Conservation of Tropical Forests in the Anthropocene.” Current Biology 29, no. 19: R1008–R1020. 10.1016/j.cub.2019.08.026. 31593660

[ece371018-bib-0045] Eklund, J. , J. P. G. Jones , M. Räsänen , et al. 2022. “Elevated Fires During COVID‐19 Lockdown and the Vulnerability of Protected Areas.” Nature Sustainability 5, no. 7: 603–609. 10.1038/s41893-022-00884-x.

[ece371018-bib-0046] Enright, N. J. , J. B. Fontaine , D. M. Bowman , R. A. Bradstock , and R. J. Williams . 2015. “Interval Squeeze: Altered Fire Regimes and Demographic Responses Interact to Threaten Woody Species Persistence as Climate Changes.” Frontiers in Ecology and the Environment 13, no. 5: 265–272. 10.1890/140231.

[ece371018-bib-0047] Falster, D. , R. Gallagher , E. H. Wenk , et al. 2021. “AusTraits, a Curated Plant Trait Database for the Australian Flora.” Scientific Data 8, no. 1: 254. 10.1038/s41597-021-01006-6. 34593819 PMC8484355

[ece371018-bib-0048] Feng, X. , C. Merow , Z. Liu , et al. 2021. “How Deregulation, Drought and Increasing Fire Impact Amazonian Biodiversity.” Nature 597, no. 7877: 516–521. 10.1038/s41586-021-03876-7. 34471291

[ece371018-bib-0049] Flores, B. M. , and M. W. Holmgren . 2021. “Sand Savannas Expand at the Core of the Amazon After Forest Wildfires.” Ecosystems 24: 1624–1637. 10.1007/s10021-021-00607-x.

[ece371018-bib-0050] Foster, C. N. , P. S. Barton , C. I. MacGregor , J. A. Catford , W. Blanchard , and D. B. Lindenmayer . 2018. “Effects of Fire Regime on Plant Species Richness and Composition Differ Among Forest, Woodland and Heath Vegetation.” Applied Vegetation Science 21, no. 1: 132–143. 10.1111/avsc.12345.

[ece371018-bib-0052] Gallagher, R. V. , S. Allen , B. D. E. Mackenzie , et al. 2021. “High Fire Frequency and the Impact of the 2019–2020 Megafires on Australian Plant Diversity.” Diversity and Distributions 27, no. 7: 1166–1179. 10.1111/ddi.13265.

[ece371018-bib-0053] Geldmann, J. , A. Manica , N. D. Burgess , L. Coad , and A. Balmford . 2019. “A Global‐Level Assessment of the Effectiveness of Protected Areas at Resisting Anthropogenic Pressures.” Proceedings of the National Academy of Sciences 116, no. 46: 23209–23215. 10.1073/pnas.1908221116. PMC6859326 31659036

[ece371018-bib-0054] Gill, A. M. , S. L. Stephens , and G. J. Cary . 2013. “The Worldwide “Wildfire” Problem.” Ecological Applications 23: 438–454. 10.1890/10-2213.1. 23634593

[ece371018-bib-0055] Giorgis, M. A. , S. R. Zeballos , L. Carbone , et al. 2021. “A Review of Fire Effects Across South American Ecosystems: The Role of Climate and Time Since Fire.” Fire Ecology 17, no. 1: 1–20. 10.1186/s42408-021-00100-9.

[ece371018-bib-0056] Gordijn, P. J. , T. M. Everson , and T. G. O'Connor . 2018. “Resistance of Drakensberg Grasslands to Compositional Change Depends on the Influence of Fire‐Return Interval and Grassland Structure on Richness and Spatial Turnover.” Perspectives in Plant Ecology, Evolution and Systematics 34: 26–36. 10.1016/j.ppees.2018.07.005.

[ece371018-bib-0057] Gorta, S. B. Z. , C. T. Callaghan , F. Samonte , et al. 2023. “Multi‐Taxon Biodiversity Responses to the 2019–2020 Australian Megafires.” Global Change Biology 29, no. 23: 6727–6740. 10.1111/gcb.16955. 37823682

[ece371018-bib-0058] Gray, C. L. , S. L. L. Hill , T. Newbold , et al. 2016. “Local Biodiversity Is Higher Inside Than Outside Terrestrial Protected Areas Worldwide.” Nature Communications 7, no. 1: 12306. 10.1038/ncomms12306. PMC4974472 27465407

[ece371018-bib-0059] Guisan, A. , and N. E. Zimmermann . 2000. “Predictive Habitat Distribution Models in Ecology.” Ecological Modelling 135: 147–186. 10.1016/S0304-3800(00)00354-9.

[ece371018-bib-0060] Hair, J. H., jr. , R. E. Anderson , R. L. Tatham , and W. C. Black . 1992. “Multiple Discriminant Analysis.” In Multivariate Data Analysis With Readings, third ed. MacMillan Publishing Company.

[ece371018-bib-0061] Hanson, J. O. 2020. “wdpar: Interface to the World Database on Protected Areas.” https://CRAN.R‐project.org/package=wdpar.

[ece371018-bib-0062] He, T. , B. B. Lamont , and J. G. Pausas . 2019. “Fire as a Key Driver of Earth's Biodiversity.” Biological Reviews 94, no. 6: 1983–2010. 10.1111/brv.12544. 31298472

[ece371018-bib-0063] Hoffmann, W. A. , E. L. Geiger , S. G. Gotsch , et al. 2012. “Ecological Thresholds at the Savanna‐Forest Boundary: How Plant Traits, Resources and Fire Govern the Distribution of Tropical Biomes.” Ecology Letters 15, no. 7: 759–768. 10.1111/j.1461-0248.2012.01789.x. 22554474

[ece371018-bib-0064] Holl, K. D. , M. E. Loik , E. H. V. Lin , and I. A. Samuels . 2000. “Tropical Montane Forest Restoration in Costa Rica: Overcoming Barriers to Dispersal and Establishment.” Restoration Ecology 8, no. 4: 339–349. 10.1046/j.1526-100x.2000.80049.x.

[ece371018-bib-0065] IPBES . 2019. “IPBES Global Assessment on Biodiversity and Ecosystem Services, Chapter 4 (Draft).”

[ece371018-bib-0066] Islas Madrid, G. E. , D. A. Rodríguez Trejo , and P. A. Martínez Hernández . 2013. “Undergrowth Diversity and Solar Radiation in a Pinus Hartwegii Lindl. Forest With Prescribed Burning Diversidad del Sotobosque y radiación Solar en Un Bosque de Pinus Hartwegii Lindl. Con Quema Prescrita.” Revista Mexicana De Ciencias Forestales 4: 25–40. http://www.scielo.org.mx/pdf/remcf/v4n15/v4n15a3.pdf.

[ece371018-bib-0067] Jauni, M. , S. Gripenberg , and S. Ramula . 2015. “Non‐native Plant Species Benefit From Disturbance: A Meta‐Analysis.” Oikos 124: 122–129. 10.1111/oik.01416.

[ece371018-bib-0068] Junior, C. , L. Aragão , M. Fonseca , C. Almeida , L. Vedovato , and L. Anderson . 2018. “Deforestation‐Induced Fragmentation Increases Forest Fire Occurrence in Central Brazilian Amazonia.” Forests 9, no. 6: 305. 10.3390/f9060305.

[ece371018-bib-0069] Kattge, J. , G. Bönisch , S. Díaz , et al. 2020. “TRY Plant Trait Database ‐ Enhanced Coverage and Open Access.” Global Change Biology 26, no. 1: 119–188. 10.1111/gcb.14904. 31891233

[ece371018-bib-0070] Kearney, S. , V. Adams , R. Fuller , H. Possingham , and J. Watson . 2020. “Estimating the Benefit of Well‐Managed Protected Areas for Threatened Species Conservation.” Oryx 54, no. 2: 276–284. 10.1017/S0030605317001739.

[ece371018-bib-0071] Keeley, J. E. , J. G. Pausas , P. W. Rundel , W. J. Bond , and R. A. Bradstock . 2011. “Fire as an Evolutionary Pressure Shaping Plant Traits.” Trends in Plant Science 16, no. 8: 406–411. 10.1016/j.tplants.2011.04.002. 21571573

[ece371018-bib-0072] Kelly, L. T. , and L. Brotons . 2017. “Using Fire to Promote Biodiversity.” Science 355, no. 6331: 1264–1265. 10.1126/science.aam7672. 28336625

[ece371018-bib-0073] Kelly, L. T. , L. Brotons , and M. A. McCarthy . 2017. “Putting Pyrodiversity to Work for Animal Conservation.” Conservation Biology 31: 952–955. 10.1111/cobi.12861. 28339129

[ece371018-bib-0074] Kelly, L. T. , K. M. Giljohann , A. Duane , et al. 2020. “Fire and Biodiversity in the Anthropocene.” Science 370, no. 6519: eabb0355. 10.1126/science.abb0355. 33214246

[ece371018-bib-0075] Kindt, R. 2020. “WorldFlora: An R Package for Exact and Fuzzy Matching of Plant Names Against the World Flora Online Taxonomic Backbone Data.” Applications in Plant Sciences 8, no. 9: e11388. 10.1002/aps3.11388. 33014632 PMC7526431

[ece371018-bib-0076] Kodandapani, N. , M. A. Cochrane , and R. Sukumar . 2004. “Conservation Threat of Increasing Fire Frequencies in the Western Ghats, India.” Conservation Biology 18, no. 6: 1553–1561. 10.1111/j.1523-1739.2004.00433.x.

[ece371018-bib-0077] Koleff, P. , K. J. Gaston , and J. K. Lennon . 2003. “Measuring Beta Diversity for Presence–Absence Data.” Journal of Animal Ecology 72: 367–382. 10.1046/j.1365-2656.2003.00710.x.

[ece371018-bib-0078] Laurance, W. , D. Carolina Useche , J. Rendeiro , et al. 2012. “Averting Biodiversity Collapse in Tropical Forest Protected Areas.” Nature 489: 290–294. 10.1038/nature11318. 22832582

[ece371018-bib-0079] Lewis, S. L. , D. P. Edwards , and D. Galbraith . 2015. “Increasing Human Dominance of Tropical Forests.” Science 349, no. 6250: 827–832. 10.1126/science.aaa9932. 26293955

[ece371018-bib-0080] Li, F. , Q. Zhu , W. J. Riley , et al. 2023. “AttentionFire_v1.0: Interpretable Machine Learning Fire Model for Burned‐Area Predictions Over Tropics.” Geoscientific Model Development 16, no. 3: 869–884. 10.5194/gmd-16-869-2023.

[ece371018-bib-0081] Liberati, A. , D. G. Altman , J. Tetzlaff , et al. 2009. “The PRISMA Statement for Reporting Systematic Reviews and Meta‐Analyses of Studies That Evaluate Health Care Interventions: Explanation and Elaboration.” PLoS Medicine 6, no. 7: e1000100. 10.1371/journal.pmed.1000100. 19621070 PMC2707010

[ece371018-bib-0082] López‐Cruz, S. D. C. , D. R. Aryal , C. A. Velázquez‐Sanabria , et al. 2022. “Effect of Prescribed Burning on Tree Diversity, Biomass Stocks and Soil Organic Carbon Storage in Tropical Highland Forests.” Forests 13: 2164. 10.3390/f13122164.

[ece371018-bib-0083] Machida, W. S. , L. Gomes , P. Moser , et al. 2021. “Long Term Post‐Fire Recovery of Woody Plants in Savannas of Central Brazil.” Forest Ecology and Management 493: 119255. 10.1016/j.foreco.2021.119255.

[ece371018-bib-0084] Maginel, C. J. , B. O. Knapp , J. M. Kabrick , and R.‐M. Muzika . 2019. “Landscape‐ and Site‐Level Responses of Woody Structure and Ground Flora to Repeated Prescribed Fire in the Missouri Ozarks.” Canadian Journal of Forest Research 49, no. 8: 1004–1014. 10.1139/cjfr-2018-0492.

[ece371018-bib-0085] Maitner, B. S. , B. Boyle , N. Casler , et al. 2017. “The Bien r Package: A Tool to Access the Botanical Information and Ecology Network (BIEN) Database.” Methods in Ecology and Evolution 9, no. 2: 373–379. 10.1111/2041-210x.12861.

[ece371018-bib-0086] McLauchlan, K. K. , P. E. Higuera , J. Miesel , et al. 2020. “Fire as a Fundamental Ecological Process: Research Advances and Frontiers.” Journal of Ecology 108, no. 5: 2047–2069. 10.1111/1365-2745.13403.

[ece371018-bib-0087] Moher, D. , A. Liberati , J. Tetzlaff , D. G. Altman , and Group. P . 2009. “Preferred Reporting Items for Systematic Reviews and Meta‐Analyses: The PRISMA Statement.” BMJ 339: b2535. 10.1136/bmj.b2535. 19622551 PMC2714657

[ece371018-bib-0088] Morrison, D. A. , G. J. Cary , S. M. Pengelly , et al. 1995. “Effects of Fire Frequency on Plant Species Composition of Sandstone Communities in the Sydney Region: Inter‐Fire Interval and Time‐Since‐Fire.” Australian Journal of Ecology 20: 239–247. 10.1111/j.1442-9993.1995.tb00535.x.

[ece371018-bib-0089] Mueller‐Dombois, D. , C. Daehler , and J. D. Jacobi . 2008. “Vegetation. Biodiversity Assessment of Tropical Island Ecosystems.” 17–48. https://www.researchgate.net/publication/266674344_Vegetation.

[ece371018-bib-0090] Nelson, Z. J. , J. W. Peter , and G. K. Stanley . 2014. “Influence of Climate and Environment on Post‐Fire Recovery of Mountain Big Sagebrush.” International Journal of Wildland Fire 23: 131–142. 10.1071/WF13012.

[ece371018-bib-0091] Nguyen, T. V. , K. J. Allen , N. C. Le , C. Q. Truong , K. Tenzin , and P. J. Baker . 2023. “Human‐Driven Fire Regime Change in the Seasonal Tropical Forests of Central Vietnam.” Geophysical Research Letters 50, no. 13: e2022GL100687. 10.1029/2022gl100687.

[ece371018-bib-0092] Nóbrega, C. C. , P. Brando , D. V. Silvério , L. Maracahipes , and P. De Marco Júnior . 2019. “Effects of Experimental Fires on the Phylogenetic and Functional Diversity of Woody Species in a Neotropical Forest.” Forest Ecology and Management 450: 117497. 10.1016/j.foreco.2019.117497.

[ece371018-bib-0093] Olson, D. , E. Dinerstein , E. Wikramanayake , et al. 2001. “Terrestrial Ecoregions of the World: A New Map of Life on Earth.” Bioscience 51: 933–938. 10.1641/0006-3568(2001)051[0933:TEOTWA]2.0.CO;2.

[ece371018-bib-0094] Pastro, L. , C. Dickman , and M. Letnic . 2011. “Burning for Biodiversity or Burning Biodiversity? Prescribed Burn vs. Wildfire Impacts on Plants, Lizards, and Mammals.” Ecological Applications 21, no. 8: 3238–3253. 10.1890/10-2351.1.

[ece371018-bib-0095] Pati, P. K. , P. Kaushik , D. Malasiya , T. Ray , M. L. Khan , and P. K. Khare . 2024. “Impacts of Forest Fire Frequency on Structure and Composition of Tropical Moist Deciduous Forest Communities of Bandhavgarh Tiger Reserve, Central India.” Trees, Forests and People 15: 100489. 10.1016/j.tfp.2023.100489.

[ece371018-bib-0096] Pausas, J. G. 2015. “Evolutionary Fire Ecology: Lessons Learned From Pines.” Trends in Plant Science 20, no. 5: 318–324. 10.1016/j.tplants.2015.03.001. 25814325

[ece371018-bib-0097] Pausas, J. G. , and W. J. Bond . 2020. “Alternative Biome States in Terrestrial Ecosystems.” Trends in Plant Science 25, no. 3: 250–263. 10.1016/j.tplants.2019.11.003. 31917105

[ece371018-bib-0098] Pausas, J. G. , and J. E. Keeley . 2019. “Wildfires as an Ecosystem Service.” Frontiers in Ecology and the Environment 17, no. 5: 289–295. 10.1002/fee.2044.

[ece371018-bib-0099] Pereira, P. , P. Mierauskas , X. Ubeda , J. Mataix‐Solera , and A. Cerdà . 2012. “Fire in Protected Areas ‐The Effect of Protection and Importance of Fire Management.” Environmental Research, Engineering and Management 59: 52–62. 10.5755/j01.erem.59.1.856.

[ece371018-bib-0100] Peterson, D. W. , and P. B. Reich . 2008. “Fire Frequency and Tree Canopy Structure Influence Plant Species Diversity in a Forest‐ Grassland Ecotone.” Plant Ecology 194: 5–16. 10.1007/s11258-007-9270-4.

[ece371018-bib-0101] Pilon, N. A. L. , M. G. B. Cava , W. A. Hoffmann , R. C. R. Abreu , A. Fidelis , and G. Durigan . 2020. “The Diversity of Post‐Fire Regeneration Strategies in the Cerrado Ground Layer.” Journal of Ecology 109, no. 1: 154–166. 10.1111/1365-2745.13456.

[ece371018-bib-0102] Pivello, V. R. 2011. “The Use of Fire in the Cerrado and Amazonian Rainforests of Brazil: Past and Present.” Fire Ecology 7, no. 1: 24–39. 10.4996/fireecology.0701024.

[ece371018-bib-0103] Plumanns‐Pouton, E. S. , M. H. Swan , T. D. Penman , L. Collins , and L. T. Kelly . 2023. “Time Since Fire Shapes Plant Immaturity Risk Across Fire Severity Classes.” Fire Ecology 19, no. 1: 25. 10.1186/s42408-023-00185-4.

[ece371018-bib-0104] Policelli, N. , P. Picca , and I. E. G. Villafañe . 2018. “Is Prescribed Fire a Suitable Management Tool to Reduce Shrub Encroachment in Palm Savannas?” Restoration Ecology 27, no. 1: 109–119. 10.1111/rec.12824.

[ece371018-bib-0105] Power, M. J. , J. Marlon , N. Ortiz , et al. 2007. “Changes in Fire Regimes Since the Last Glacial Maximum: An Assessment Based on a Global Synthesis and Analysis of Charcoal Data.” Climate Dynamics 30, no. 7–8: 887–907. 10.1007/s00382-007-0334-x.

[ece371018-bib-0106] Probert, J. R. , C. L. Parr , R. M. Holdo , et al. 2019. “Anthropogenic Modifications to Fire Regimes in the Wider Serengeti‐Mara Ecosystem.” Global Change Biology 25, no. 10: 3406–3423. 10.1111/gcb.14711. 31282085 PMC6852266

[ece371018-bib-0107] R Core Development Team . 2024. “R: A Language and Environment for Statistical Computing.” R Foundation for Statistical Computing, Vienna, Austria. https://www.R‐project.org/.

[ece371018-bib-0108] Roces‐Díaz, J. V. , C. Santín , J. Martínez‐Vilalta , and S. H. Doerr . 2021. “A Global Synthesis of Fire Effects on Ecosystem Services of Forests and Woodlands.” Frontiers in Ecology and the Environment 20, no. 3: 170–178. 10.1002/fee.2349.

[ece371018-bib-0109] Rodrigues, C. A. , and A. Fidélis . 2022. “Should We Burn the Cerrado? Effects of Fire Frequency on Open Savanna Plant Communities.” Journal of Vegetation Science 33, no. 6: 13159. 10.1111/jvs.13159.

[ece371018-bib-0110] Rodríguez‐Trejo, D. A. , and P. Z. Fulé . 2003. “Fire Ecology of Mexican Pines and a Fire Management Proposal.” International Journal of Wildland Fire 12, no. 1: 23. 10.1071/wf02040.

[ece371018-bib-0111] Schielzeth, H. 2010. “Simple Means to Improve the Interpretability of Regression Coefficients.” Methods in Ecology and Evolution 1: 103–113. 10.1111/j.2041-210X.2010.00012.x.

[ece371018-bib-0112] Schmidt, I. B. , L. C. Moura , M. C. Ferreira , et al. 2018. “Fire Management in the Brazilian Savanna: First Steps and the Way Forward.” Journal of Applied Ecology 55, no. 5: 2094–2101. 10.1111/1365-2664.13118.

[ece371018-bib-0113] Sheehan, T. , D. Bachelet , and K. Ferschweiler . 2019. “Fire, CO2, and Climate Effects on Modeled Vegetation and Carbon Dynamics in Western Oregon and Washington.” PLoS One 14, no. 1: e0210989. 10.1371/journal.pone.0210989. 30682107 PMC6347276

[ece371018-bib-0114] Shepherd, H. E. R. , J. A. Catford , M. N. Steele , et al. 2021. “Propagule Availability Drives Post‐Wildfire Recovery of Peatland Plant Communities.” Applied Vegetation Science 24: e12608. 10.1111/avsc.12608.

[ece371018-bib-0115] Simon, M. F. , and T. Pennington . 2012. “Evidence for Adaptation to Fire Regimes in the Tropical Savannas of the Brazilian Cerrado.” International Journal of Plant Sciences 173, no. 6: 711–723. 10.1086/665973.

[ece371018-bib-0116] Smit, I. P. J. , G. P. Asner , N. Govender , T. Kennedy‐Bowdoin , D. Knapp , and J. Jacobson . 2010. “Effects of Fire on Woody Vegetation Structure in African Savanna.” Ecological Applications 20, no. 7: 1865–1875. 10.1890/09-0929.1. 21049875

[ece371018-bib-0117] Socolar, J. B. , J. J. Gilroy , W. E. Kunin , and D. P. Edwards . 2016. “How Should Beta‐Diversity Inform Biodiversity Conservation?” Trends in Ecology & Evolution 1: 67–80. 10.1016/j.tree.2015.11.005. 26701706

[ece371018-bib-0118] Sørensen, T. A. 1948. “A Method of Establishing Groups of Equal Amplitude in Plant Sociology Based on Similarity of Species Content, and Its Application to Analyses of the Vegetation on Danish Commons.” Kongelige Danske Videnskabernes Selskabs. Biologiske Skrifter 5: 1–34.

[ece371018-bib-0119] Staver, A. C. , S. Archibald , and S. Levin . 2011. “Tree Cover in Sub‐Saharan Africa: Rainfall and Fire Constrain Forest and Savanna as Alternative Stable States.” Ecology 92, no. 5: 1063–1072. 10.1890/10-1684.1. 21661567

[ece371018-bib-0120] Tingley, M. , V. Ruiz‐Gutierrez , R. Wilkerson , C. Howell , and R. Siegel . 2016. “Pyrodiversity Promotes Avian Diversity Over the Decade Following Forest Fire.” Proceedings of the Royal Society B: Biological Sciences 283, no. 1840: 20161703. 10.1098/rspb.2016.1703. PMC5069516 27708152

[ece371018-bib-0121] Tyre, A. 2017. “Does Model Averaging Make Sense?” Online blog of Dr Andrew Tyre. http://atyre2.github.io/2017/06/16/rebutting_cade.html.

[ece371018-bib-0122] Tyukavina, A. , P. Potapov , M. C. Hansen , et al. 2022. “Global Trends of Forest Loss due to Fire From 2001 to 2019.” Frontiers in Remote Sensing 3: 825190. 10.3389/frsen.2022.825190.

[ece371018-bib-0123] UNEP‐WCMC & IUCN . 2020. Protected Planet: The World Database on Protected Areas (WDPA) and World Database on Other Effective Area‐Based Conservation Measures (WD‐OECM) [Online]. UNEP‐WCMC and IUCN. www.protectedplanet.net.

[ece371018-bib-0124] UNFCC . 2019. “REDD+ Web platform.” https://redd.unfccc.int/.

[ece371018-bib-0125] Verma, S. , D. Singh , A. K. Singh , and S. Jayakumar . 2019. “Post‐Fire Soil Nutrient Dynamics in a Tropical Dry Deciduous Forest of Western Ghats, India.” Forest Ecosystems 6, no. 1: 6. 10.1186/s40663-019-0168-0.

[ece371018-bib-0126] Williamson, G. J. , D. Y. P. Tng , and D. M. J. S. Bowman . 2024. “Climate, Fire, and Anthropogenic Disturbance Determine the Current Global Distribution of Tropical Forest and Savanna.” Environmental Research Letters 19, no. 2: 24032. 10.1088/1748-9326/ad20ac.

[ece371018-bib-0127] Wimberly, M. C. , D. Wanyama , R. Doughty , H. Peiro , and S. Crowell . 2024. “Increasing Fire Activity in African Tropical Forests Is Associated With Deforestation and Climate Change.” Geophysical Research Letters 51, no. 9: e2023GL106240. 10.1029/2023gl106240.

[ece371018-bib-0128] Wu, C. , S. Venevsky , S. Sitch , L. M. Mercado , C. Huntingford , and A. C. Staver . 2021. “Historical and Future Global Burned Area With Changing Climate and Human Demography.” One Earth 4, no. 4: 517–530. 10.1016/j.oneear.2021.03.002.

