# Supplementary material for: A Pantropical Analysis of Fire Impacts and Post‐Fire Species Recovery of Plant Life Forms
Source: Ecol Evol. 2025 Feb 17;15(2):e71018. doi: 10.1002/ece3.71018 (PMC11832907; doi:10.1002/ece3.71018)
Supplement: Supplementary file 1 — Appendix S1. [file ECE3-15-e71018-s003.docx]

**Appendix 1**

**List of studies used in the data.**

1. ADEMOH, F. O., MUOGHALU, J. I. & ONWUMERE, B. 2017. Temporal pattern of tree community dynamics in a secondary forest in southwestern Nigeria, 29 years after a ground fire. *Global Ecology and Conservation,* 9**,** 148-170.
2. BOUGHTON, E. H., BOHLEN, P. J. & STEELE, C. 2013. Season of fire and nutrient enrichment affect plant community dynamics in subtropical semi-natural grasslands released from agriculture. *Biological Conservation,* 158**,** 239-247.
3. CLEARY, D. F. R. & PRIADJATI, A. 2005. Vegetation responses to burning in a rain forest in Borneo. *Plant Ecology,* 177**,** 145-163.
4. DE OLIVEIRA, M. T., DAMASCENO-JUNIOR, G. A., POTT, A., PARANHOS FILHO, A. C., SUAREZ, Y. R. & PAROLIN, P. 2014. Regeneration of riparian forests of the Brazilian Pantanal under flood and fire influence. *Forest Ecology and Management,* 331**,** 256-263.
5. DOUGLAS, M. M., SETTERFIELD, S. A., MCGUINNESS, K. & LAKE, P. S. 2015. The impact of fire on riparian vegetation in Australia's tropical savanna. *Freshwater Science,* 34**,** 1351-1365.
6. FORD, A. J. & HARDESTY, B. D. 2012. Species adaptation to both fire and climate change in tropical montane heath: Can Melaleuca uxorum (Myrtaceae) survive? Pacific Conservation Biology, 18, 319-324.
7. GALVAO DE MELO, A. C. & DURIGAN, G. 2010. Fire impact and dynamics of plant community regeneration at the seasonal semideciduous forest edge (Galia, SP, Brazil). Revista Brasileira de Botanica, 33, 37-50.
8. GOMES, L., MARACAHIPES, L., MARIMON, B. S., REIS, S. M., ELIAS, F., MARACAHIPES-SANTOS, L., MARIMON-JUNIOR, B. H. & LENZA, E. 2014. Post-fire recovery of savanna vegetation from rocky outcrops. Flora, 209, 201-208.
9. HERRERA, A.H., BALLERA, B.L.G.B., TORO-MANRÍQUEZ, M.D.R., LENCINAS, M.V., MARTÍNEZ PASTUR, G.J. & RAMÍREZ, H.H. 2021. Changes in Vegetation of Flooded Savannas Subject to Cattle Grazing and Fire in Plains of Colombia. Land, 10(2), p.108.
10. ISLAS MADRID, G. E., RODRÍGUEZ TREJO, D. A. & MARTÍNEZ HERNÁNDEZ, P. A. 2013. Undergrowth diversity and solar radiation in a Pinus hartwegii Lindl. forest with prescribed burning Diversidad del sotobosque y radiación solar en un bosque de Pinus hartwegii Lindl. con quema prescrita. Revista mexicana de ciencias forestales, 4, 25-40.
11. JANCOSKI, H. S., PINTO, J. R. R., NOGUEIRA, D. S., MEWS, H. A., ABAD, J. C. S., SCALON, M. C. & MARIMON, B. S. 2019. Fine-scale effects of fire on non-woody species in a southern Amazonian seasonal wetland. Wetlands Ecology and Management, 27, 267-281.
12. JHARIYA, M. K., BARGALI, S. S., SWAMY, S. L. & KITTUR, B. 2012. Vegetational Structure, Diversity and Fuel Load in Fire Affected Areas of Tropical Dry Deciduous Forests in Chhattisgarh. Vegetos, 25, 210-224.
13. LE STRADIC, S., HERNANDEZ, P., FERNANDES, G. W. & BUISSON, E. 2018. Regeneration after fire in campo rupestre: Short- and long-term vegetation dynamics. Flora, 238, 191-200.
14. LEWIS, T., REIF, M., PRENDERGAST, E. & CUONG, T. 2012. The effect of long-term repeated burning and fire exclusion on above- and below-ground Blackbutt (Eucalyptus pilularis) forest vegetation assemblages. Austral Ecology, 37, 767-778.
15. LU, P.-L. & DELAY, J. K. 2016. Vegetation and fire in lowland dry forest at Wa'ahila Ridge on O'ahu, Hawai'i. PhytoKeys, 51-64.
16. MATAYAYA, G., WUTA, M. & NYAMADZAWO, G. 2017. Effects of different disturbance regimes on grass and herbaceous plant diversity and biomass in Zimbabwean dambo systems. International Journal of Biodiversity Science Ecosystem Services & Management, 13, 181-190.
17. MCKENNA, P., ERSKINE, P. D., GLENN, V. & DOLEY, D. 2019. Response of open woodland and grassland mine site rehabilitation to fire disturbance on engineered landforms. *Ecological Engineering,* 133**,** 98-108.
18. MUDONGO, E., FYNN, R. & BONYONGO, M. C. 2016. Influence of fire on woody vegetation density, cover and structure at Tiisa Kalahari Ranch in western Botswana. *Grassland Science,* 62**,** 3-11.
19. NEWMAN, E. A., WINKLER, C. A. & HEMBRY, D. H. 2018b. Effects of anthropogenic wildfire in low-elevation Pacific island vegetation communities in French Polynesia. Peerj, 6.
20. NOVAK, E.N., BERTELSEN, M., DAVIS, D., GROBERT, D.M., LYONS, K.G., MARTINA, J.P., MCCAW, W.M., O’TOOLE, M. & VELDMAN, J.W. 2021. Season of prescribed fire determines grassland restoration outcomes after fire exclusion and overgrazing. Ecosphere, 12(9).
21. PETTIT, N. E. & NAIMAN, R. J. 2007. Postfire response of flood-regenerating riparian vegetation in a semi-arid landscape. Ecology, 88, 2094-2104.
22. ROCHA, M., SANTOS JUNIOR, C. C., DAMASCENO-JUNIOR, G. A., POTT, V. J. & POTT, A. 2015. Effect of fire on a monodominant floating mat of Cyperus giganteus Vahl in a neotropical wetland. Brazilian Journal of Biology, 75, 114-124.
23. RUSSELL-SMITH, J., RYAN, P. G. & CHEAL, D. C. 2002. Fire regimes and the conservation of sandstone heath in monsoonal northern Australia: frequency, interval, patchiness. Biological Conservation, 104, 91-106.
24. SALAZAR, A. & GOLDSTEIN, G. 2014. Effects of Fire on Seedling Diversity and Plant Reproduction (Sexual vs. Vegetative) in Neotropical Savannas Differing in Tree Density. Biotropica, 46, 139-147.
25. SANTANA, L.D., RIBEIRO, J.H.C., VAN DEN BERG, E. & CARVALHO, F.A. 2020. Impact on soil and tree community of a threatened subtropical phytophysiognomy after a forest fire. Folia Geobotanica, 55(2), pp.81–93.
26. SILVA, L.S. DA, COSTA, T.R., SALOMÃO, N.V., OTONI, T.J.O. & MACHADO, E.L.M. 2020. After-fire Variations in Floristic Composition at the Cerrado (Brazilian Savannah) Phytophysiognomies in Curvelo, Minas Gerais, Brazil. Floresta e Ambiente, 27(3).
27. WARD, B. G., BRAGG, T. B. & HAYES, B. A. 2018. Effects of chaining and burning in Acacia ramulosa shrublands of the Peron Peninsula, Shark Bay, Western Australia. International Journal of Wildland Fire, 27, 623-635.
28. WOINARSKI, J. C. Z., RISLER, J. & KEAN, L. 2004. Response of vegetation and vertebrate fauna to 23 years of fire exclusion in a tropical Eucalyptus open forest, Northern Territory, Australia. Austral Ecology, 29, 156-176.
