# Supplementary material for: A Pantropical Analysis of Fire Impacts and Post‐Fire Species Recovery of Plant Life Forms
Source: Ecol Evol. 2025 Feb 17;15(2):e71018. doi: 10.1002/ece3.71018 (PMC11832907; doi:10.1002/ece3.71018)
Supplement: Supplementary file 4 — Appendix S4. [file ECE3-15-e71018-s004.docx]

**Appendix 4**

**Table S3**

Plant groups and their representation in the number of studies, sites, and observations in protected vs non-protected areas and biome types.

|  | Fire-adaptive | | Fire -sensitive | |  |
| --- | --- | --- | --- | --- | --- |
|  | Protected | Non-protected | Protected | Non-protected | **Total** |
| **Trees/Shrubs** | | | | | |
| **No of studies** | 7 | 9 | 6 | 4 | **26** |
| **No of sites** | 14 | 28 | 32 | 10 | **84** |
| **Observations** | 373 | 760 | 2019 | 383 | **3535** |
| **Forbs** | | | | | |
| **No of studies** | 6 | 7 | 3 | 4 | **20** |
| **No of sites** | 13 | 33 | 7 | 8 | **61** |
| **Observations** | 175 | 545 | 41 | 37 | **798** |
| **Graminoids** | | | | | |
| **No of studies** | 5 | 7 | 3 | 3 | **18** |
| **No of sites** | 10 | 33 | 6 | 7 | **56** |
| **Observations** | 165 | 384 | 61 | 41 | **651** |
| **Climbers** | | | | | |
| **No of studies** | 6 | 8 | 5 | 5 | **24** |
| **No of sites** | 12 | 25 | 13 | 10 | **60** |
| **Observations** | 47 | 130 | 40 | 56 | **273** |
