# Supplementary material for: A Pantropical Analysis of Fire Impacts and Post‐Fire Species Recovery of Plant Life Forms
Source: Ecol Evol. 2025 Feb 17;15(2):e71018. doi: 10.1002/ece3.71018 (PMC11832907; doi:10.1002/ece3.71018)
Supplement: Supplementary file 5 — Appendix S5. [file ECE3-15-e71018-s001.docx]

Appendix 5

**Table S4**

**Candidate models.**

Response variable either the Relative Species Richness or the Species Turnover (Sorenson Index).

| Model | Equation |
| --- | --- |
| M00 | 1 + (1\|study_ID) |
| M01 | Time Since Fire + (1\|study_ID) |
| M02 | Fire Type + (1\|study_ID) |
| M03 | Biome Type + (1\|study_ID) |
| M04 | Protection Status+ (1\|study_ID) |
| M05 | Time Since Fire + Fire Type + (1\|study_ID) |
| M06 | Time Since Fire +Biome Type + (1\|study_ID) |
| M07 | Time Since Fire +Protection Status+ (1\|study_ID) |
| M08 | Fire Type + Biome Type + (1\|study_ID) |
| M09 | Fire Type + Protection Status+ (1\|study_ID) |
| M10 | Biome Type +Protection Status+ (1\|study_ID) |
| M11 | Time Since Fire +Fire Type+ Biome Type + (1\|study_ID) |
| M12 | Time Since Fire +Fire Type+ Protection Status+ (1\|study_ID) |
| M13 | Time Since Fire +Biome Type + Protection Status+ (1\|study_ID) |
| M14 | Fire Type + Biome Type + Protection Status+ (1\|study_ID) |
| M15 | Time Since Fire +Fire Type + Biome Type + Protection Status+(1\|study_ID) |
| M16 | Time Since Fire +Biome Type + Time Since Fire*Biome Type + (1\|study_ID) |
| M17 | Time Since Fire + Protection status+ Time Since Fire*Protection Status+ (1\|study_ID) |
| M18 | Fire Type + Biome Type + Fire Type*Biome Type + (1\|study_ID) |
| M19 | Fire Type+ Protection Status+ Fire Type*Protection Status+ (1\|study_ID) |
| M20 | Fire Type+ Time Since Fire*Biome Type + (1\|study_ID) |
| M21 | Time Since Fire +Fire Type*Biome Type + (1\|study_ID) |
| M22 | Fire Type+ Time Since Fire*Protection Status+ (1\|study_ID) |
| M23 | Time Since Fire + Fire Type*Protection Status+(1\|study_ID) |
| M24 | Protection status+ Time Since Fire*Biome Type +(1\|study_ID) |
| M25 | Biome Type + Time Since Fire*Protection Status+ (1\|study_ID) |
| M26 | Protection status+ Fire Type*Biome Type + (1\|study_ID) |
| M27 | Biome Type + Fire Type*Protection Status+ (1\|study_ID) |
| M28 | Fire Type + Protection status+ Time Since Fire*Biome Type + (1\|study_ID) |
| M29 | Time Since Fire +Fire Type + Time Since Fire*Protection Status+ (1\|study_ID) |
| M30 | Time Since Fire + Protection status+ Fire Type*Biome Type + (1\|study_ID) |
| M31 | Time Since Fire +Biome Type +Fire Type*Protection Status+ (1\|study_ID) |
